# Phasic pupillary responses reveal differential engagement of attentional control in bilingual spoken language processing

**DOI:** 10.1038/s41598-021-03008-1

**Published:** 2021-12-06

**Authors:** Anne L. Beatty-Martínez, Rosa E. Guzzardo Tamargo, Paola E. Dussias

**Affiliations:** 1 Department of Psychology, McGill University, Montréal, QC H3A1G1 Canada; 2 Department of Hispanic Studies, University of Puerto Rico-Río Piedras, San Juan, PR 00931 USA; 3 Department of Spanish, Italian and Portuguese, Pennsylvania State University, University Park, PA 16802 USA

**Keywords:** Attention, Cognitive control, Language

## Abstract

Language processing is cognitively demanding, requiring attentional resources to efficiently select and extract linguistic information as utterances unfold. Previous research has associated changes in pupil size with increased attentional effort. However, it is unknown whether the behavioral ecology of speakers may differentially affect engagement of attentional resources involved in conversation. For bilinguals, such an act potentially involves competing signals in more than one language and how this competition arises may differ across communicative contexts. We examined changes in pupil size during the comprehension of unilingual and codeswitched speech in a richly-characterized bilingual sample. In a visual-world task, participants saw pairs of objects as they heard instructions to select a target image. Instructions were either unilingual or codeswitched from one language to the other. We found that only bilinguals who use each of their languages in separate communicative contexts and who have high attention ability, show differential attention to unilingual and codeswitched speech. Bilinguals for whom codeswitching is common practice process unilingual and codeswitched speech similarly, regardless of attentional skill. Taken together, these results suggest that bilinguals recruit different language control strategies for distinct communicative purposes. The interactional context of language use critically determines attentional control engagement during language processing.

## Introduction

Language processing is cognitively demanding, requiring attentional resources to efficiently select and modulate goal-relevant information^1^. In everyday conversation, listeners must selectively attend to natural speech in the presence of competing signals to extract linguistic information as utterances unfold. Depending on the nature of the conversation, listeners may also need to adjust their breadth of attentional focus to attend to broader or narrower events^2,3^. Distinct attentional states can coexist. In overview, we distinguish between those associated with the processing of a given topic content (e.g., following instructions) from those associated with processes of language control (e.g., whether one or multiple languages are used^4^). In contrast to monolingual speakers, whose language environment is generally considered to be relatively homogeneous and unilingual^5^, bilinguals are often immersed in a complex and linguistically diverse environment in which multiple languages are involved even within the same conversation. In consequence, bilinguals must adapt to detect critical features that discriminate one language from another so as to assess the appropriateness of using one, the other, or both^6,7^.

Recent theoretical perspectives posit that the flexibility and efficiency to which bilinguals draw on attentional resources associated with language control is mediated in part by the interactional demands of the language environment^4,8–10^. As such, even bilinguals who are highly proficient in both languages may show different patterns of adaptive response depending on their habits of language use: whether they use each language in separate communicative contexts, whether they habitually codeswitch making use of more than one language opportunistically, and whether others with whom they interact are similarly bilingual^9,11,12^. We follow Green and colleagues^4,10^ in supposing that bilinguals can adopt different modes of language control subject to their socio-pragmatic appropriateness. In a context where just one language is in use, language control is engaged competitively, requiring a narrow focus of attention to exploit the resources of the target language. Conversely, in a context that involves more than one language, language control may be coordinated cooperatively. Here, language membership is minimized, inducing a broader attentional state in which resources of both language networks are explored.

For present purposes, we focus on codeswitching contexts where most speakers opportunistically use and frequently switch between languages in everyday life. Codeswitching is often described as the hallmark of bilingualism, yet remains relatively understudied with respect to how language and attentional control processes are engaged^11,12^. Language switching within artificial paradigms (e.g., tasks in which bilinguals are asked to switch between languages in response to exogeneous cues such as a color displayed on a computer screen^13^) have been shown to engage conflict monitoring brain regions such as the prefrontal and anterior cingulate cortices^14–16^; conversely and critically, naturalistic codeswitching does not recruit these areas^6^ nor does it seem to have a strong association to a particular domain-general control strategy^17–20^. Notwithstanding, if codeswitching contexts involve a cooperative as opposed to a competitive relation between the two languages, then one possibility is that differences in codeswitching experience may modulate the way bilinguals engage attentional control during language processing.

Some evidence supports this conjecture. Beatty-Martínez and Dussias^21^ examined electrophysiological responses to unilingual and codeswitched sentences in two groups of highly proficient Spanish–English bilinguals who differed in codeswitching experience. One group lived in Spain and rarely switched between languages in conversation. The other group was comprised of habitual codeswitchers living in the United States. For non-codeswitching and codeswitching bilinguals alike, no differences in processing were found between Spanish and English unilingual translation equivalent sentences. Notwithstanding, the ERP record revealed differences in the way the two groups processed codeswitched sentences. Non-codeswitching bilinguals consistently showed a larger early frontal positivity to codeswitching, indicating that they can reliably detect a change in language at early stages of processing, presumably due to their proven experience at maximizing language competition. This contrasts with the results from codeswitching bilinguals, who did not show such differences, suggesting that when language control is engaged cooperatively, and therefore, both languages are expected, bilinguals are able to maintain a sufficiently broad focus of attention when processing codeswitches. More recent research has corroborated these results and has found that bilinguals can shift between competitive and cooperative language control states, showing a modulation of switch effects as a function of the social context (i.e., by the co-presence of a bilingual or monolingual interlocutor^22^).

In the present study, we aim to determine indices of bilinguals’ language control states by examining changes in pupil size during the comprehension of unilingual and codeswitched speech. As an indicator of activity of the locus coeruleus-norepinephrine (LC-NE) system, pupil dilation has been associated with the tradeoff between exploration and exploitation of attentional control^23–26^. This tradeoff is related to changes in the balance of tonic and phasic neuronal firing mode of LC activity, where increased tonic activation is associated with task disengagement and exploration (e.g., mind wandering^27^), while increased phasic activation is associated with stimulus-dependent changes in attention and effort (e.g., simultaneous interpretation^28^). LC activity is highly plastic and driven by higher-cortical conflict-monitoring regions, such as the anterior cingulate and orbitofrontal cortices^24^. When faced with high conflict demands, these regions relay conflict signals that promote greater task engagement and exploitation, resulting in robust phasic pupillary responses. Of note, previous research suggests that individuals with high attentional abilities can more optimally regulate LC activity to perform successfully, and thus exhibit larger increases in pupil size associated with greater conflict^29^. As such, changes in pupil size provide a promising lens to examine individual differences in information-seeking behaviors.

Pupillometry is becoming increasingly popular in studies of spoken language processing, due to its high ecological validity for tracking dynamic changes of attentional control. To date, a large number of studies has shown that, in general, larger pupil responses are associated with increasing attentional demands related to linguistic uncertainty and complexity^30,31^. For instance, McCloy et al.^32^ observed that switching attention between two competing speech streams led to an increase in pupil size relative to focusing attention on one speaker. Relative to monolinguals, bilinguals exhibit a greater pupil response when processing in their second (L2) language^33,34^. Large pupil sizes have also been observed for bilinguals when listening or speaking in their L2 compared to their L1^28,35^, although these effects appear to be attenuated by language and experiential factors such as cognate status of words^36^ and individuals’ linguistic proficiency^37^.

Relatively less is known about how pupil size changes as function of switching between languages. In a seminal study, Hyönä et al.^28^ examined the effects of task demands on pupil size in skilled interpreters. Pupillary responses were compared across three conditions in which participants had to listen to, shadow, or simultaneously interpret short passages or single words. Pupil responses were larger for the L2, especially for words that did not have a one-word translation equivalent in the L1. They also found that pupil responses varied across tasks, with the largest pupil dilation for simultaneous interpretation when compared with listening and shadowing conditions. More recently, Byers-Heinlein et al.^38^ examined pupil responses in bilingual infants and adults living in Montréal, Canada. Using the visual-world paradigm, participants saw pairs of pictures (e.g., a dog and a book), and heard either a unilingual (“Look! Find the dog!”) or a codeswitched (“Look! Find *le chien*!”) sentence naming one of the pictures. Compared to unilingual sentences, codeswitched sentences were associated with larger pupil responses, indicating that switching languages is more effortful. Importantly, this difference was attenuated when switches were more predictable, such as those occurring in natural breakpoints in speech (for converging magnetoencephalographic evidence, see^6^). Notably, these findings speak to the degree of cognitive effort and language control involved in switching between languages. Together, this body of work shows that the pupillary response is sensitive to a variety of language-related processes and can therefore provide valuable insights into the way in which bilingual listeners selectively attend to and extract linguistic information on the fly.

What remains unclear is whether pupil responses to language processes, particularly those associated with a language switch, are differentially modulated by individual differences in attentional control abilities and/or depending on bilinguals’ habits of language use. Previous research has shown that better attentional control skill is associated with better speech processing and larger pupil responses in relatively challenging speech processing conditions^39–41^. Moreover, Kuchinsky et al.^42^ observed that individuals who showed larger pupil dilation exhibited greater activity in the right primary auditory cortex and were better able to sustain and adapt attentional focus during task switching. Therefore, one possibility is that individuals with higher attentional abilities may be able to regulate fluctuations in LC activity during codeswitching more optimally^23^.

In the current study, we investigate two individual difference factors that may influence pupil responses, but which have not been jointly examined: auditory attentional control and *language cooperativeness,* operationalized here as the tendency to use more than one language in conversation. Specifically, we make use of the elevator counting with reversal subset of the Test of Everyday Attention^43^ (TEA), an ecological measure of auditory attentional control while also paying close attention to the community context of language use. As alluded to earlier, we propose that the way in which bilinguals draw on attentional resources associated with language control will depend on bilinguals’ habits of language use and the control demands of their interactional context^9,10,17,21^. To this end, we focus on Spanish–English bilinguals from Puerto Rico, a predominantly Spanish-speaking environment but where English is loosely supported with little-to-no interactional cost and where codeswitching is very common^12,17,44^. Codeswitching experience sits on a continuum that is influenced by conventionalized communicative norms^12,21,45,46^ but also varies according to sociodemographic and individual characteristics^47^. We therefore treat the construct of language cooperativeness as a continuous measure to evaluate language control states as a function of bilinguals’ habits of language use.

Based on the evidence just reviewed, we might expect a more robust pupil response to codeswitch relative to unilingual conditions as reflecting the effort involved in processing a change in language^28,38^. Notwithstanding, we hypothesize that any processing costs associated with codeswitching would be modulated by individual differences in attention ability and language cooperativeness. Building on the association between cognitively demanding attentional control tasks and pupil responses^31^, we predict that individuals with high attentional control ability will show a greater difference between unilingual and codeswitched conditions, such that those with better performance on the TEA task will have larger pupils after hearing a codeswitch relative to its unilingual equivalent. Furthermore, if pupillary responses index distinct language control states^4,10^, we should expect the processing of codeswitched speech to be modulated by the ways in which bilinguals use their languages in conversation. Specifically, for individuals who tend to use their languages more competitively (i.e., using each language in separate communicative contexts), codeswitches may signal a processing conflict, requiring greater allocation of attentional resources by shifting LC activity toward increased phasic activation^23^. Notwithstanding, if codeswitching experience is associated with a more cooperative language control state^4,8,21^ and does not recruit conflict monitoring regions that drive phasic LC activity^6^, then we can further predict that differences in pupil responses between unilingual and codeswitch conditions will be attenuated or eliminated with increasing language cooperativeness.

## Results

**Figure 1:**
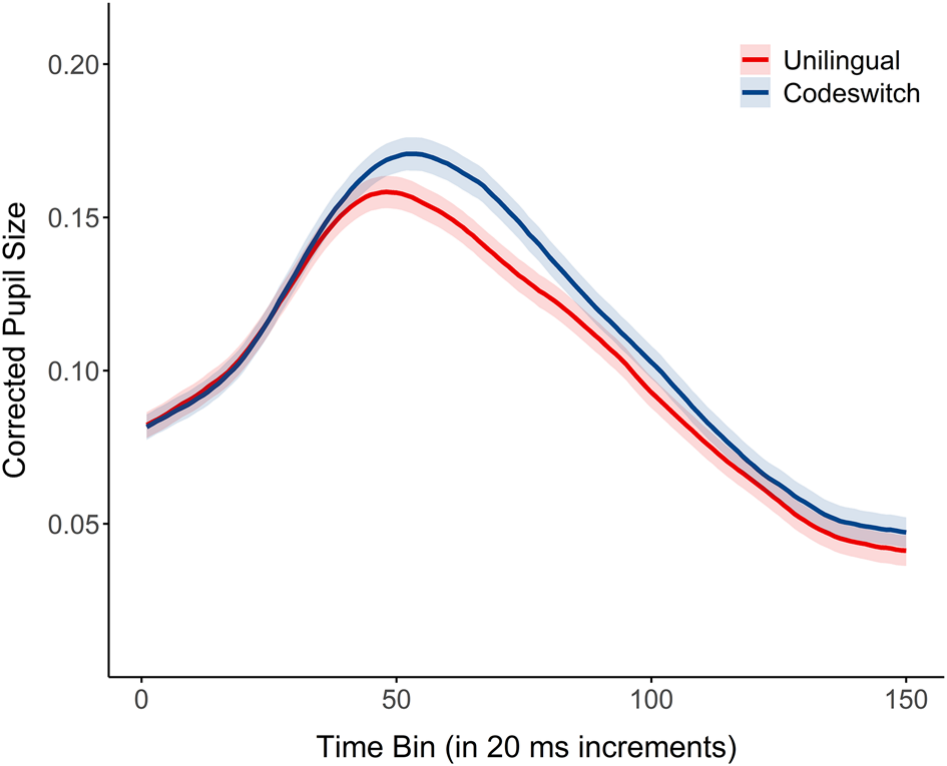
Corrected pupil size for unilingual Spanish (red) and codeswitch (blue) conditions. Shading represents standard error of the mean.

Descriptives for mouse click data are available in Supplementary Table S1. The pupil size corresponding to unilingual and codeswitch conditions is shown in Fig. 1. Our analysis examined the influence of auditory attention ability and language cooperativeness on pupil size during the processing of unilingual and codeswitched speech. The estimated parameters of the final generalized additive mixed model are found in Table 1. This analysis revealed a significant nonlinear interaction surface between time, attention ability, and language cooperativeness ratings for both unilingual and codeswitched conditions. The summary statistics of the full model fitted with the ordered factor difference surface further revealed that the interaction surface significantly differed between unilingual and codeswitched conditions (*Edf* = 24.09, *F* = 2.89, *p* < .001; full model specification and output summary, see Supplementary Table S2).

**Table 1:** Generalized additive mixed model reporting parametric coefficients and estimated degrees of freedom (Edf), reference degrees of freedom (Ref.df), F-values, and p-values for the tensor product, smooth term, and random effects.

Parametric coefficients	Estimate	SE	t-value	*p* value
(Intercept)	.09	.09	9.96	< .001
LangCS	.01	.00	1.16	0.24

Smooth terms	Edf	Ref.df	F-value	*p* value
te(Time,Attention,LangCoop):LangSpa	18.29	20.1	9.50	< .001
te(Time,Attention,LangCoop):LangCS	43.14	54.0	5.29	< .001
s(Xgaze,Ygaze)	7.88	10.8	12.59	< .001
s(Time,Participant)	319.75	910.0	2.49	< .001
s(Time,Item)	209.44	1819.0	0.32	< .001

Statistical significance was further assessed using two complementary strategies (for suggested guidelines, see^48–50^). The first criterion involved using an fREML-based model comparison between the full model and a nested model without the interactions of both parametric and smooth terms for language. In this approach, fixed effects are estimated as random effects, which validates performing likelihood ratio tests on models fitted with fREML^49^. Chi-square test of fREML scores indicated that the full model was preferred over the nested model (*X*^2^(5) = 11.66, *p* < .001). The second criterion involved re-specifying the model with a binary difference tensor by creating a dummy variable (IsCS) where codeswitch is equal to 1 and unilingual Spanish is set as 0 (i.e., the reference level; for full model specification and output summary, see Supplementary Table S3). This approach essentially integrates parametric and smooth difference terms, allowing us to evaluate whether the non-linear interactions differ significantly between unilingual Spanish and codeswitch conditions in a single confirmatory test. Results from the binary difference model indicated that the nonlinear interaction surface significantly differed between unilingual and codeswitch conditions (*Edf* = 25.10, *F* = 2.99, *p* < .001).

**Figure 2:**
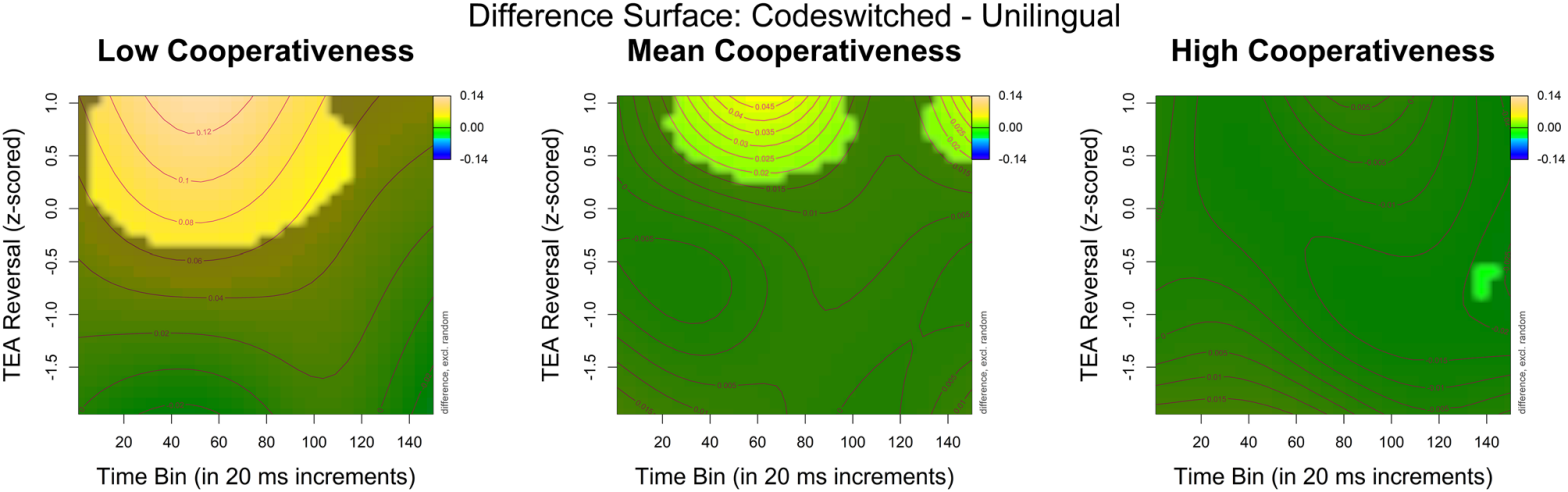
Contour plots of the interaction between time, attention ability, and the difference in pupil size between unilingual Spanish and codeswitch conditions at different values of language cooperativeness. Time (in 20 ms bins) is plotted on the x-axis. Z-standardized scores on the TEA reversal subtest (where higher scores indicate better performance) are plotted on the y-axis. The panels presented represent language cooperativeness ratings at the minimum, the mean, and the maximum value. Brighter yellows indicate a larger difference in pupil size for codeswitch relative to unilingual conditions while darker blues indicate the opposite. The contour lines represent the model-predicted difference in pupil size values with highlighted areas indicating the region(s) in the surface that are significantly different from zero. An animation of the full interaction is provided as Supplementary Video S1.

In interpreting generalized additive mixed models, visual inspection of the model’s estimates is essential. Figure 2 displays the effect of the interaction between time, attention ability, and language cooperativeness on the difference in pupil size between unilingual and codeswitch conditions. To best characterize this interaction, it is presented as a multi-panel plot showing the contour surfaces of the estimated difference in pupil size (codeswitch minus unilingual) across time and attention ability at three values of language cooperativeness, namely the minimum, the mean, and the maximum. An animation of the full interaction is provided as Supplementary Video S1. In the visualization, brighter yellows indicate a larger difference in pupil size for codeswitch relative to unilingual conditions while darker blues indicate the opposite. The contour lines represent the model-predicted difference in pupil size values with highlighted areas indicating the region(s) in the surface that are significantly different from zero. Based on these surface plots, we can make the following observations. At low levels of language cooperativeness (i.e., those individuals who reported using just one language in conversation), there is an effect of auditory attention ability such that only individuals with high accuracy on the TEA reversal subtest exhibited larger pupil responses for codeswitch relative to unilingual Spanish conditions. This effect progressively attenuates and ultimately disappears with increasing language cooperativeness. Thus, irrespective of attentional skill, bilinguals who reported a greater tendency to use more than one language in conversation did not show this effect. Taken together, these results suggest that when language control is engaged cooperatively, bilinguals can maintain a sufficiently broad focus of attention when processing a codeswitch.

## Discussion

The overarching goal of this study was to determine how codeswitching experience differentially modulates engagement of attentional resources to unilingual and codeswitched speech. To do so, we capitalized on recent theoretical frameworks positing that distinct language control attentional states are mediated by the interactional demands of the language environment. By studying Spanish–English bilinguals from Puerto Rico, where the two languages are well-supported and codeswitching is prevalent, this work aimed to dissociate between competitive and cooperative modes of language control by examining changes in pupillary responses during bilingual language processing. Our results revealed that only bilinguals who reported a greater tendency to make use of their languages competitively (i.e., in separate communicative contexts) and who had high attention ability, showed differential engagement of attentional control as indexed by larger increases in pupil size during the comprehension of codeswitched speech. This finding provides converging evidence for the proposal that competitive language control requires a narrow attentional control state to avoid the distraction of the language not in use^4,21,22^. Bilinguals whose recurrent habits of conversational exchanges are typically unilingual (i.e., characterized by relatively low language entropy^51^) must critically maximize language competition within a conversation, and as such, must allocate greater effort upon hearing a codeswitch to exploit the new target language. This contrasts with the results from bilinguals reporting a greater tendency to make use of their languages cooperatively, who did not show such differences, regardless of attentional control skill. We interpreted these findings as evidence that when language control is engaged cooperatively, bilinguals can explore functionally distinct language networks requiring a broader attentional state^21^. The implication is that codeswitching is not inherently effortful, and that bilingual language processing will depend on how bilinguals make use of their languages as shaped by the control demands of their interactional context.

Whereas past research suggested that switching languages is effortful, placing high demands on control processes, it is becoming increasingly clear that there are boundary conditions to previously-observed effects of codeswitching^6,12,21,52–55^. This emerging body of work suggests that bilinguals who habitually codeswitch in their daily life tend to process naturalistic codeswitches in a manner akin to unilingual speech. The results of the current study are consistent with this claim and provide further insight into the mechanisms that underlie language switching more generally. Studies on bilingual language control have long been interested in identifying the nature of switch costs using both voluntary and cued language switching tasks^56,57^. If distinct attentional states mediate different types of language use with implications for language processing, then this argument requires us to not only richly characterize individuals in terms of their habits of language use (and, by extension, the interactional context at the site of testing)^11,17,58^, but also specify the immediate context and attentional control demands of the experiment proper^59^. To this end, it is imperative to show how different interactional experiences relate to different paths of adaptive change in language control processes and to frame research questions in ways that reveal and exploit the boundary conditions on such variability.

Our results revealed that, even in contexts where codeswitching is a regular communicative practice, the processing of codeswitched speech depends, in part, on bilinguals’ habits of language use, namely the communicative demands of their recurrent interactional exchanges (i.e., if they typically tend to use just one or more languages in conversation). This point has important implications for the interpretation of the pupil response as an indicator of distinct language control states. Specifically, it means that a larger pupil response to switching may be viewed as signaling an increase demand for control for bilinguals who tend to keep their languages separately, presumably due to their experience of needing to implicitly monitor and control the non-target language. This interpretation is neurobiologically plausible as evidenced by the link between changes in pupil diameter and coordinated activity between the LC and the anterior cingulate cortex^60,61^. This explanation may also account for why we only observed the effect of attention ability at lower levels of language cooperativeness. Thus, low cooperative bilinguals with high attention abilities may come to regulate between-language competition by more optimally focusing on the target language while ignoring competing information from the other. Intriguingly, there is magnetoencephalographic (MEG) evidence on codeswitching bilinguals that listening to naturalistic codeswitched speech (as opposed to externally-cued language switching paradigms) does not increase activity in anterior cingulate networks^6^. Thus, one exciting possibility is that bilinguals’ habits of language use mediate differential gating of signals associated with language control^4,10^. Specifically, we propose that high cooperative language use shapes a gating system that flexibly allows attention to be broadened so that resources from either language may be explored. Although this explanation is indeed speculative, future work should explore these links through the co-registration of MEG and pupil data.

An alternative explanation to competitive and cooperative language control states may be envisaged: bilinguals with high language cooperativeness have greater codeswitching experience, increasing the predictability of a codeswitch overall. However, codeswitch and unilingual trials were evenly distributed throughout the experiment to ensure the equality and certainty for both language conditions. More importantly, even in contexts where codeswitching is common practice, the vast majority of utterances bilinguals produce are unilingual (e.g., 5.8% of utterances in the Bangor-Miami corpus^62^). Given this, unilingual utterances should be relatively more predictable over and above codeswitched ones for all bilinguals, despite differences in codeswitching experience. An important future direction will be to examine pupil responses to codeswitching in situations that require competitive language control (e.g., in monolingual settings where switching languages comes at a high interactional cost). If changes in pupil size are related to the predictability of codeswitches, then we should expect differences between unilingual and codeswitch conditions to decrease with greater codeswitching experience. Notwithstanding, if the effects reported here are instead related to differences in attentional states, then we should expect bilinguals to adopt a more competitive language control mode, thereby restricting access to the target language irrespective of their level of language cooperativeness (e.g., Kaan et al.^22^). Thus, future work could expand on the current findings by examining how individuals move between cooperative and competitive control states, thereby further elucidating how language processing may differ even within the same individual under different conditions^59^.

What about bilinguals with low values of cooperativeness and low attention ability? Recall that for these individuals, as for those with high values of cooperativeness, pupil size was unaffected by the presence of a codeswitch. This raises the question of how different pathways can lead to the same outcome^63^. One possibility is that such individuals inadvertently had more momentary lapses in attention during the task and simply ignored signals of language change. Indeed, it has been shown that individuals’ attentional engagement may fluctuate over the course of an experiment due to distraction or mind wandering, which in turn has consequences for language processing^64^. One way to test whether this is the case is to track changes in oscillatory activity in the alpha band to examine fluctuations in attention during listening comprehension^65^. Specifically, we would expect increased alpha power to be associated with lower attention ability and similar pupil dilations between unilingual and codeswitched conditions. In this way, examining the role of individual differences in pupillary response to unilingual and codeswitched speech offers a potential way to explore degeneracy in bilingual language control^58,66^. Further work is needed to identify and characterize more precisely the factors and pathways that underlie adaptive change.

In this study, we have demonstrated that codeswitching experience differentially modulates attentional control engagement during bilingual spoken language processing. Critically, these findings would not be apparent had we simply examined differences in pupil responses without considering the joint effect of individual attentional skill and bilinguals’ habits of language use. Taken together, these data highlight the value of examining interactions over main effects^67^ and overall provide a compelling case for the need for a rich characterization of the participant sample and their interactional context^11,58^ coupled with ecologically valid measures of codeswitching^53,68^. Although the pattern of results is complex, it provides important insight into the flexibility and efficiency to which bilinguals draw on resources associated with language control as they selectively attend to and extract linguistic information on the fly.

## Methods

### Participants

A total of 100 Spanish–English bilinguals were recruited at the University of Puerto Rico, Río Piedras. All participants gave informed consent, and the procedures had the approval of The Pennsylvania State University Institutional Review Board (Approval Number: 34810). All research was performed in accordance with the relevant guidelines and regulations set forth by the same review board. Participants were paid $10/hr for their participation. One participant was excluded from the sample after reporting being born and predominantly raised outside of Puerto Rico, in a monolingual Spanish environment. Seven additional participants’ data were excluded due to data collection errors or insufficient items per condition (i.e., following outlier removal), leaving a total of 92 (26 male) participants.

Participants completed a customized web-based questionnaire that asked them to describe their bilingual language experience. All participants were native Spanish speakers who acquired Spanish at birth and English either simultaneously or in early childhood, and all reported high levels of proficiency in both languages. We calculated participants’ language cooperativeness based on the item ‘I tend to use more than one language in conversation with certain people” rated on a 9-point scale with responses from “never” to “always”. This item was repeated across three distinct communicative contexts (i.e., at home, at school, and during leisure time) but exemplified differently for each context (e.g., when speaking with family members, with some colleagues, and with some friends). Participants’ ratings did not differ across communicative contexts. For these reasons, we calculated language cooperativeness as the average value across the three contexts. Here, lower values indicate greater compartmentalization of language use (i.e., Spanish and English are used in separate and distinct communicative contexts; the relevance of language membership is maximized). Conversely, higher values indicate greater integrative use of both languages (i.e., Spanish and English are used cooperatively/opportunistically; the relevance of language membership is minimized). We note that this construct differs from other recent measures examining diversity of language use such as language entropy^51^ and general indexes of codeswitching frequency^18^, in that it focuses on bilinguals’ language choices when interacting with interlocutors and not across distinct communicative contexts more broadly. Participant characteristics are summarized in Table 2.

**Table 2:** Participant self-reported characteristics.

Measure	*M*	*SD*	*95% CI*
Age, years	20.5	2.11	[20.8, 21.0]
AoA: Spanish	0.53	1.29	[0.3, 0.8]
AoA: English	3.89	2.35	[3.4, 4.4]
Proficiency: Spanish (/10)	9.32	0.97	[9.1, 9.5]
Proficiency: English (/10)	9.18	0.76	[9.0, 9.3]
Language cooperativeness (/9)	7.50	1.71	[7.2, 7.9]

Means, standard deviations, and 95% CIs for age, age of acquisition (AoA), proficiency self-ratings, and language cooperativeness measures are shown. Proficiency self-ratings were made on a 10-point scale ranging from 1 (less) to 10 (more). Self-ratings for language cooperativeness were made on a 9-point scale ranging from 1 (never) to 9 (always). All values represent raw, non-standardized scores.

To provide an objective measure of Spanish and English proficiency, participants performed a semantic category fluency task in both languages. Participants were asked to generate as many exemplars as possible that belong to a semantic category within a 30-s time limit. Written instructions indicating the language to be used appeared on the screen, and the order of language of production was blocked such that participants completed four categories in Spanish first, and four different categories in English second. The categories were counterbalanced across language blocks and participants, such that each category was presented in each language block, but no participant completed the same category in both languages. Performance was analyzed by calculating the total number of exemplars produced across categories in Spanish and in English. These scores revealed high and relatively balanced verbal abilities in Spanish (*M* = 43.5, *SD* = 6.89; *95% CI* [42.1, 44.9]) and English (*M* = 43.1, *SD* = 6.03; *95% CI* [41.9, 44.4]). Furthermore, a correlational analysis revealed a positive association between Spanish in English (*r*(90) = 0.43, *p* < .001, *95% CI* [0.25, 0.59]), further reflecting the interdependent use of the two languages.

In addition to the verbal fluency tasks, participants completed the elevator counting subset from the Test of Everyday Attention (TEA) battery^43^. The subset comprises a series of tasks designed to measure distinct attentional components using ecologically-valid stimuli that relate to everyday situations. The counting subtest assesses sustained attention. Participants had to imagine that they were in an elevator where the floor indicator sign was not working. For each trial, participants had to determine which floor they have arrived at by counting the number of tones presented at a moderately slow pace and at irregular intervals. The distraction subtest assesses auditory selective attention. Using the same elevator analogy, participants were asked to count low-pitched tones (as presented during the counting subtest), while ignoring interspersed high-pitched tones. The reversal subtest assesses attentional switching between two unpredictable directions of counting. Participants were presented with three types of tones (high-pitched, middle-pitched, and low-pitched). They were asked to count the middle-pitched tones upwards if preceded by a high-pitched tone or downwards if preceded by a low-pitched tone. Together, the three subtests took approximately 20 min to complete. Performance on each subtest was measured as the percentage of trials with correct responses (see Table 3). A near-ceiling effect was observed in the elevator counting subtest, confirming that participants had normal hearing. Participants were overall less accurate on the elevator distraction and reversal subtests. Our analysis focused solely on the elevator counting with reversal subtest (henceforth TEA-Reversal), where participants exhibited greater variability (for other studies on bilingualism examining this subtest, see^69,70^). TEA-Reversal accuracy was not significantly correlated with language cooperativeness (*r*(89) = − 0.08, *p* = .442, *95% CI* [− 0.28, 0.13]).

**Table 3:** Proportion of correct responses in elevator subtests from the TEA battery.

Measure	*M*	*SD*	*95% CI*
TEA-Counting	.98	.07	[.96, .99]
TEA-Distraction	.75	.27	[.70, .81]
TEA-Reversal	.65	.33	[.58, .71]

Means, standard deviations, and 95% CIs for scores on elevator counting, distraction, and reversal subtests from the Test of Everyday Attention (TEA) battery.

### Stimuli

#### Visual Stimuli

Visual materials for the pupillometry experiment were retrieved from the online database #Soyvisual (http://www.soyvisual.org; released under Creative Common license BY-NC-SA). All chromatic information was removed by converting all images to grayscale. An independent group of 34 Spanish–English bilinguals from the University of Puerto Rico, Río Piedras was recruited to name the selected stimuli in both languages. Items were included if naming agreement was 70% or higher in each language. Using these criteria, 184 photographs were selected for experimental trials. The photographs were sorted into 92 target-distractor pairs (a full list of experimental stimuli is included in Supplementary Table S4) matched for visual complexity (*t* = 0.28, *p* = 0.778), and semantic category (e.g., animals, food, etc.).

#### Auditory stimuli

For the corresponding sound files, experimental stimuli were embedded in one of two carrier phrases: “Encuentra el” (English: Find the_MASC_), for all masculine target nouns and “Encuentra la” (English: Find the_FEM_), for all feminine target nouns. The gender of the definite determiner always agreed with the gender of the target noun (or the gender of its Spanish translation equivalent for codeswitch conditions). For both unilingual Spanish and codeswitch conditions alike, the determiner and target agreed with the gender of the distractor noun on half of the trials. Auditory stimuli were recorded by a native Puerto Rican female speaker in a sound-attenuated room using a Fostex DC-R302 recorder and a head-mounted Audix HT5 condenser microphone. The speaker read both variants of the carrier phrase (i.e., “Encuentra el”, “Encuentra la”) several times in a clear but natural style. All words were recorded separately to avoid the possibility of coarticulation effects. The full set of Spanish targets were recorded first, followed by the full set of English targets, repeating each target at least three times. All stimuli were manipulated in the same way using Audacity (© Audacity Team) with a 44.1 kHz sampling rate, ensuring that any effects could not be due to differences in acoustic manipulation across conditions. Sound files were subsequently segmented and annotated manually using the Praat software for phonetic analysis^71^. From these, the clearest token was selected for each word in the carrier phrase. The duration for *encuentra* was 1280 ms, and the duration for both *el* and *la* was 500 ms each. A 100 ms pause was inserted between *encuentra* and each determiner. Thus, determiner onset was always at 1380 ms after the onset of the carrier phrase. For each noun, a single token was selected among the speaker’s productions and spliced onto its corresponding carrier phrase. A 50 ms pause was inserted at the offset of the determiner. Therefore, the onset of target words always occurred at 1930 ms after the onset of the carrier phrase.

#### Target word characteristics

Target-distractor pairs were counterbalanced such that each item appeared as a target and as a distractor and appeared equally often in unilingual Spanish and codeswitch conditions across experimental lists following a Latin square design. A mirror version of each experimental list was also created to counterbalance the on-screen position of the target (i.e., each target appeared equally either on the left- or right-hand position of the monitor). The 184 pairs of target words (i.e., the target word in both languages corresponding to each photograph; e.g., tenedor/fork) were matched for naming agreement (*t*(317.62) = − 1.25, *p* = 0.213) and lexical frequency (*t*(350.23) = 0.99, *p* = 0.324). Additionally, target and distractor words were all non-cognates and did not share initial phonemes in either language. Target word characteristics are provided in Supplementary Table S5.

### Procedure

**Figure 3:**
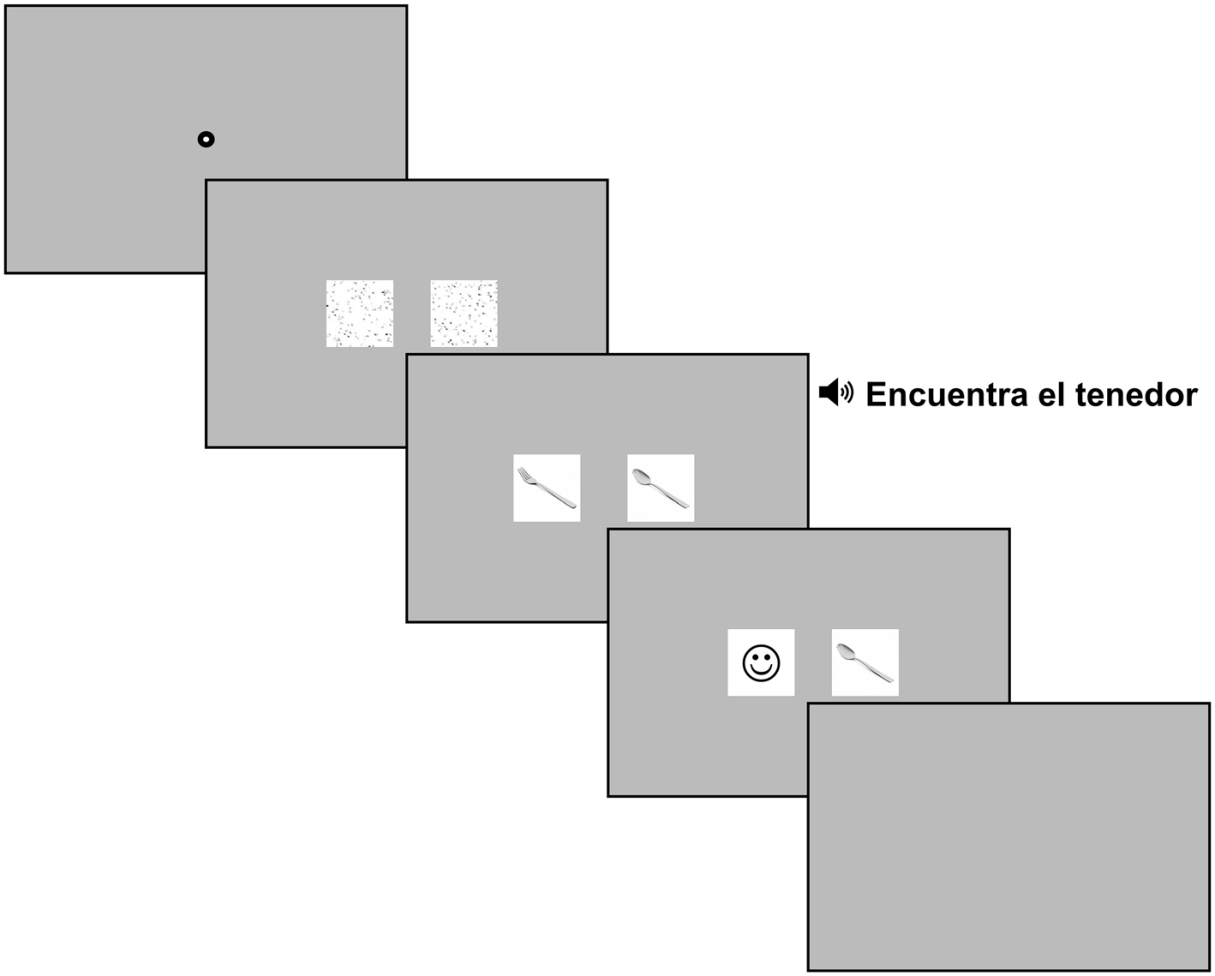
Sample trial procedure for the visual world experiment. Each trial started with a fixation point, followed by the presentation of the scrambled versions of target and distractor stimuli (1000 ms). Next, the unscrambled target and distractor images appeared while participants heard “Encuentra el/la [target].” (English: Find the_MASC/FEM_ [target].). Target and distractor images had been on the screen for 2430 ms at the onset of the target noun. Participants had to then click on one of the target object as quickly and accurately as possible, and they received feedback after their response was recorded. A delay period of 3000 ms was concatenated at the end of the target word to allow the pupil to return to baseline. Pictures remained onscreen until the end of the delay period. Each trial ended with a blank gray screen.

The experimental procedure consisted of two sessions and took place in a quiet and dimly lit room. Each of the two sessions began first with informed consent. In the first session, participants performed the pupillometry experiment. Participants were seated comfortably in a stable chair behind a chinrest set in front of the eye-tracker and the computer monitor. Pupil data were recorded from participants’ right eye at 1000 Hz using an Eyelink 1000 eye tracker (SR Research, Ottawa, Canada). The experiment was displayed on a 21-inch Viewsonic G225f. CRT monitor, which was set approximately 70 cm from the chin rest. For each participant, the height of the chinrest was adjusted to a comfortable position and to ensure that participants’ eye level aligned with the center of the screen. At the beginning of the experiment, a standard nine-point calibration and validation procedure was performed in which participants were asked to fixate on a black dot that appeared randomly on a 3 × 3 grid. Once calibration was completed, participants were given instructions on the experimental task. The experiment was divided into two blocks with a short break between blocks. Calibration procedures were repeated at the start of each experimental block and throughout the experiment as needed. Every block started with four practice trials followed pseudorandomized 92 experimental and 32 filler trials. The order of the blocks was counterbalanced between participants. At the start of each trial, a black fixation dot appeared on a gray background in the center of screen to direct participants’ eyes towards the center of the screen. To control for luminance effects, scrambled versions of each photograph were constructed by randomly re-arranging 5 × 5 pixel blocks. These were presented 1000 ms before the unscrambled photographs appeared on the screen. Participants were told that on each trial, they would first briefly see two abstract images followed by two photographs of objects displayed in the middle of the computer screen. They would then hear instructions to find a specific object. Their task was to click on the correct object as quickly and accurately as possible. Participants had up to 3000 ms to click on one of the objects and they received feedback after their response was recorded. Each trial ended with a blank gray screen. Figure 3 illustrates the trial design for the visual world task. In the second session, participants completed the language background questionnaire, the category verbal fluency task, and the TEA subtests. Each experimental session lasted approximately 40 to 60 min.

### Pupillometry analysis

#### Data processing

Pupil data were extracted using SR Research DataViewer (v.4.1.63). The baseline interest period began 1000 ms prior to noun onset, and the target interest period began at noun onset and continued for 3000 ms. Following the recommendations of van Rij et al.^72^, the data were downsampled to 50 Hz (i.e., 20 ms time bins) and normalized by subtracting the average pupil size in the baseline period from the average pupil size in each bin of the target interest period for each trial. Trials where the total proportion of samples that occurred in a blink or saccade event or outside of the central interest area exceeded 25% in either the baseline or target interest periods were excluded from further analysis, resulting in 14.01% of trials being excluded.

#### Data analysis

Normalized pupil size was modeled as a time-dependent variable using generalized additive mixed-effects models (GAMMs) as implemented in the mgcv^73^ (v. 1.8.36) and itsadug^74^ (v. 2.472) packages in the R statistical software environment^75^ (v. 4.0.274). GAMMs differ from linear mixed effect regression models in that they can additionally represent non-linear patterns (i.e., smooth terms) in data as the sum of a set of mathematical basis functions. GAMMs are particularly useful for the analysis of pupil data as they allow to explore potential nonlinear interactions between the pupil response, experimental, and individual difference predictors while also accounting for non-linear random effects and autocorrelation inherent in time-series data^72^.

Our set of analyses examined how individual differences in attentional control and codeswitching experience relate to differences in pupil size during bilingual language processing. The full model included time bin, language (two levels: unilingual or codeswitch), standardized TEA-Reversal accuracy scores, and standardized language cooperativeness ratings as a four-way interaction using a tensor product, which allows the smooth coefficients for one variable to vary non-linearly depending on the value of the other variable. Additionally, an isometric non-linear interaction between X- and Y-gaze coordinates was included to account for sudden increases and drops in pupil size due to changes in gaze position^72^. For random effects, by-participant and by-item factor smooths for time bin were included to allow for the shape of the average time course to vary by participant and item. To decrease the probability of committing a Type I and II error, fixed smooth terms were constructed using thin plate regression splines, while random smooth terms were constructed using cubic regression splines as basis functions (for power simulations, see^49^). To enhance computational efficiency, smoothing parameters were estimated using the fast-restricted maximum likelihood (fREML) method with discretized covariates^48,49^ (discrete = TRUE). Goodness-of-fit was assessed using the mgcv::gam.check function^73^. Because the distribution of the residuals exhibited heavier tails rather than a normal distribution, the model was refitted using a scaled-t family which transforms the residuals back to normality^50,76,77^. Moreover, patterns of autocorrelation in the residuals were estimated using the itsadug::acf_resid function and corrected using a first-order autoregressive AR(1) error model^48,50,72^. To evaluate the difference between unilingual and codeswitch contours, we converted the language predictor to an ordered factor with contrast coding (reference level: unilingual) and extracted the estimated p-values for the parametric and smooth difference terms^48–50^. Using difference terms in the model allows us to explicitly test whether pupil responses differ between unilingual and codeswitched conditions^50^. Data code and output summaries for additional models are available in the Open Science Framework repository: https://osf.io/8msvk/.

## Supplementary Information

Supplementary Information 1.

Supplementary Video S1.

## Data Availability

The data analyzed for this study are included in this published article as a supplementary information file.

## Code availability

The datasets generated during the current study are available in the Open Science Framework repository: https://osf.io/8msvk/.
