# Supplementary material for: Phasic pupillary responses reveal differential engagement of attentional control in bilingual spoken language processing
Source: Sci Rep. 2021 Dec 6;11:23474. doi: 10.1038/s41598-021-03008-1 (PMC8648769; doi:10.1038/s41598-021-03008-1)
Supplement: Supplementary file 1 — Supplementary Information 1. [file 41598_2021_3008_MOESM1_ESM.docx]

**Supplementary Information for**

Phasic pupillary responses reveal differential engagement of attentional control in bilingual spoken language processing

Anne L. Beatty-Martínez^*^, Rosa E. Guzzardo Tamargo & Paola E. Dussias

*Corresponding author: Anne L. Beatty-Martínez.

**Email:**  [anne.beatty-martinez@mail.mcgill.ca](mailto:anne.beatty-martinez@mail.mcgill.ca)

**This PDF file includes:**

Tables S1 to S5

Legend for Video S1

SI References

**Other supplementary materials for this manuscript include the following:**

Video S1

Supplementary Information

**Results.**

Table S1. Accuracy and reaction time for mouse click data.

| **Variable** | **M** | **SD** | **95% CI** |
| --- | --- | --- | --- |
| Unilingual Spanish accuracy | 99.5 | 2.5 | [99.0, 100] |
| Codeswitch accuracy | 99.5 | 2.6 | [98.9, 100] |
| Unilingual Spanish latency (*ms*) | 3427 | 208 | [3384, 3470] |
| Codeswitch latency (*ms*) | 3449 | 197 | [3408, 3490] |

Accuracy was at ceiling (>99%) for both unilingual and codeswitch conditions and was not analyzed further. Mouse click response times (RTs) from correct trials were calculated from target onset. Extreme outliers were excluded through visual inspections using histograms and boxplots. A paired *t* test on log-transformed RTs revealed no significant differences between unilingual and codeswitch conditions (*t*(91) = 1.6, *p* = 0.113).

Table S2. Model with an ordered factor difference smooth.

| **Parametric coefficients** | **Estimate** | **SE** | **t-value** | **p-value** |
| --- | --- | --- | --- | --- |
| (Intercept) | .09 | .01 | 10.10 | < .001 |
| LangOrdCS | .00 | .01 | 0.70 | 0.48 |
| **Smooth terms** | **Edf** | **Ref.df** | **F-value** | **p-value** |
| te(Time,Attention,Cooperativeness) | 37.37 | 41.9 | 8.38 | < .001 |
| te(Time,Attention,Cooperativeness):LangOrdCS | 24.09 | 29.2 | 2.89 | < .001 |
| s(Xgaze,Ygaze) | 7.96 | 10.9 | 12.59 | < .001 |
| s(Time,Participant) | 300.61 | 902.0 | 2.25 | < .001 |
| s(Time,Item) | 210.34 | 1818.0 | 0.32 | < .001 |
| Formula: Pupil ~ LanguageOrd + te(Time, Attention, Cooperativeness, bs = "tp") + te(Time, Attention, Cooperativeness, bs = "tp", by = LanguageOrd) + + s(Xgaze, Ygaze) + s(Time, Participant, bs = "fs", m = 1, xt = list(bs = "cr")) + s(Time, Item, bs = "fs", m = 1, xt = list(bs = "cr")) | | | | |

Table S3. Model with a binary difference smooth.

| **Parametric coefficients** | **Estimate** | **SE** | **t-value** | **p-value** |
| --- | --- | --- | --- | --- |
| (Intercept) | .09 | .01 | 10.1 | < .001 |
| **Smooth terms** | **Edf** | **Ref.df** | **F-value** | **p-value** |
| te(Time,Attention,Cooperativeness) | 37.36 | 41.9 | 8.38 | < .001 |
| te(Time,Attention,Cooperativeness):IsCS | 25.10 | 30.2 | 2.99 | < .001 |
| s(Xgaze,Ygaze) | 7.96 | 10.9 | 12.59 | < .001 |
| s(Time,Participant) | 300.69 | 902.0 | 2.26 | < .001 |
| s(Time,Item) | 210.36 | 1818.0 | 0.33 | < .001 |
| Formula: Pupil ~ + te(Time, Attention, Cooperativeness, bs = "tp") + te(Time, Attention, Cooperativeness, bs = "tp", by = IsCS) + s(Xgaze, Ygaze) + s(Time, Participant, bs = "fs", m = 1, xt = list(bs = "cr")) + s(Time, Item, bs = "fs", m = 1, xt = list(bs = "cr")) | | | | |

Video S1 (separate file). Difference surface contour animation plot of the interaction between time, attention ability on the difference in pupil size between unilingual Spanish and codeswitch conditions at different values of language cooperativeness. The panels presented represent standardized language cooperativeness ratings ranging from the minimum to the maximum value in .2 increments. Brighter yellows indicate a larger difference in pupil size for codeswitch relative to unilingual conditions while darker blues indicate the opposite. The contour lines represent the model-predicted difference in pupil size values with highlighted areas indicating the region(s) in the surface that are significantly different from zero.

**Materials.**

Table S4. Stimuli for pupillometry experiment (filler trials not included). Photographs were retrieved from the online database #Soyvisual ([www.soyvisual.org](http://www.soyvisual.org)) which are part of a collective work owned by the Orange Foundation created under a Creative Commons license (BY-NC-SA).

| forest/bosque | sun/sol | river/río | fire/fuego |
| --- | --- | --- | --- |
| moon/luna | waterfall/cascada | beach/playa | cloud/nube |
| sunflower/girasol | tree/árbol | fish/pescado | pepper/pimiento |
| palmtree/palma | leaf/hoja | butter/mantequilla | lettuce/lechuga |
| garlic/ajo | egg/huevo | rice/arroz | chicken/pollo |
| potato/papa | onion/cebolla | carrot/zanahoria | eggplant/berenjena |
| gum/chicle | bread/pan | cake/bizcocho | ice-cream/helado |
| apple/manzana | pumpkin/calabaza | lollipop/paleta | honey/miel |
| cheese/queso | avocado/aguacate | poison/veneno | wine/vino |
| pineapple/piña | orange/china | beer/cerveza | milk/leche |
| oven/horno | toilet/inodoro | dresser/gavetero | bench/banco |
| fridge/nevera | door/puerta | washer/lavadora | crib/cuna |
| nail/clavo | screw/tornillo | screwdriver/destornillador | brush/pincel |
| key/llave | feather/pluma | ruler/regla | flashlight/linterna |
| knife/cuchillo | fan/abanico | drawing/dibujo | envelope/sobre |
| broom/escoba | shovel/pala | compass/brújula | notebook/libreta |
| pen/bolígrafo | sharpener/sacapuntas | whistle/pito | gift/regalo |
| can/lata | eraser/goma | shower/ducha | box/caja |
| cup/vaso | chess/ajedrez | sink/fregadero | newspaper/periódico |
| printer/impresora | chair/silla | teapot/tetera | basket/canasta |
| mirror/espejo | puzzle/rompecabezas | knot/nudo | bucket/cubo |
| carpet/alfombra | window/ventana | candle/vela | ladder/escalera |
| hairbrush/cepillo | rocket/cohete | hammer/martillo | pencil/lápiz |
| doll/muñeca | ball/pelota | kite/chiringa | tie/corbata |
| helmet/casco | airplane/avión | underwear/calzoncillo | dress/traje |
| skateboard/patineta | bus/guagua | towel/toalla | bracelet /pulsera |
| trash/zafacón | balloon/globo | book/libro | glove/guante |
| comb/peinilla | suitcase/maleta | skirt/falda | table/mesa |
| keyboard/teclado | alarm clock/despertador | fan/abanico | briefcase/maletín |
| stapler/grapadora | dumbbell/pesa | jump rope/cuica | iron/plancha |
| hanger/gancho | watch/reloj | soap/jabón | fork/tenedor |
| lightbulb/bombilla | umbrella/sombrilla | spoon/cuchara | earring/pantalla |
| lock/candado | wire/alambre | drum/tambor | mouse/ratón |
| rope/soga | glass/copa | bag/bolsa | pillow/almohada |
| snail/caracol | shark/tiburón | pig/cerdo | bird/pájaro |
| butterfly/mariposa | pigeon/paloma | ant/hormiga | squirrel/ardilla |
| owl/buho | mouse/ratón | crab/cangrejo | rooster/gallo |
| sheep/oveja | parrot/cotorra | seal/foca | whale/ballena |
| rabbit/conejo | lizard/lagartijo | octopus/pulpo | bull/toro |
| chicken/gallina | spider/araña | snake/serpiente | cow/vaca |
| foot/pie | arm/brazo | eye/ojo | hair/pelo |
| hand/mano | leg/pierna | back/espalda | nose/nariz |
| finger/dedo | ankle/tobillo | neck/cuello | shoulder/hombro |
| knee/rodilla | wrist/muñeca | mouth/boca | tongue/lengua |
| pacifier/bobo | hat/sombrero | shoe/zapato | diaper/pañal |
| sock/media | t-shirt/camiseta | scarf/bufanda | razor/navaja |

Table S5. Target word characteristics.

| \|  \| **Spanish Targets** \| \| **English Targets** \| \| \| --- \| --- \| --- \| --- \| --- \| \|  \| *M* \| *SD* \| *M* \| *SD* \| \| Naming accuracy \| 0.89 \| 0.15 \| 0.87 \| 0.14 \| \| Lexical frequency \| 1.06 \| 0.57 \| 1.12 \| 0.61 \|   **Note:** Naming accuracy is based on norming data. Word frequency values for both languages were obtained using the CLEARPOND database^1^. Frequencies are given as logarithm to base 10. |
| --- | --- | --- | --- | --- | --- | --- | --- | --- | --- | --- | --- | --- | --- | --- | --- | --- | --- | --- | --- | --- |

**SI References**

1. Marian, V., Bartolotti, J., Chabal, S. & Shook, A. CLEARPOND: Cross-linguistic easy-access resource for phonological and orthographic neighborhood densities. *PLoS One* **7**, (2012).
